# Shape representations in the primate dorsal visual stream

**DOI:** 10.3389/fncom.2015.00043

**Published:** 2015-04-22

**Authors:** Tom Theys, Maria C. Romero, Johannes van Loon, Peter Janssen

**Affiliations:** ^1^Laboratorium voor Neuro- en Psychofysiologie, Katholieke Universiteit LeuvenLeuven, Belgium; ^2^Afdeling Experimentele Neurochirurgie en Neuroanatomie, Katholieke Universiteit LeuvenLeuven, Belgium

**Keywords:** object, shape, visual cortex, macaque, depth, parietal cortex, dorsal stream

## Abstract

The primate visual system extracts object shape information for object recognition in the ventral visual stream. Recent research has demonstrated that object shape is also processed in the dorsal visual stream, which is specialized for spatial vision and the planning of actions. A number of studies have investigated the coding of 2D shape in the anterior intraparietal area (AIP), one of the end-stage areas of the dorsal stream which has been implicated in the extraction of affordances for the purpose of grasping. These findings challenge the current understanding of area AIP as a critical stage in the dorsal stream for the extraction of object affordances. The representation of three-dimensional (3D) shape has been studied in two interconnected areas known to be critical for object grasping: area AIP and area F5a in the ventral premotor cortex (PMv), to which AIP projects. In both areas neurons respond selectively to 3D shape defined by binocular disparity, but the latency of the neural selectivity is approximately 10 ms longer in F5a compared to AIP, consistent with its higher position in the hierarchy of cortical areas. Furthermore, F5a neurons were more sensitive to small amplitudes of 3D curvature and could detect subtle differences in 3D structure more reliably than AIP neurons. In both areas, 3D-shape selective neurons were co-localized with neurons showing motor-related activity during object grasping in the dark, indicating a close convergence of visual and motor information on the same clusters of neurons.

## Introduction

**Figure 1:**
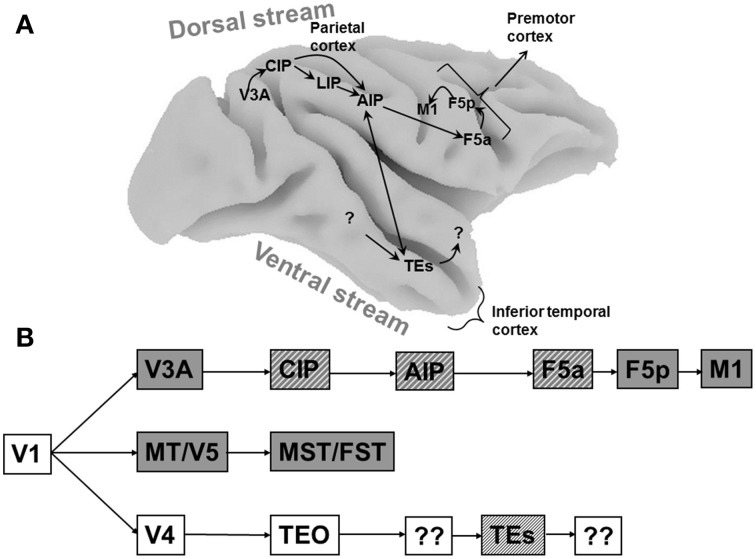
**Cortical areas processing object shape. (A)** Overview of the macaque brain illustrating the locations of the areas involved in processing object shape, and the most important connections between these areas. Unidirectional arrows indicate the presumed flow of visual information along the dorsal stream, the bidirectional arrow between AIP and TEs indicates that the direction of information flow is unclear at present. Note that most connections in extrastriate cortex are bidirectional. CIP, caudal intraparietal area; LIP, lateral intraparietal area; AIP, anterior intraparietal area; F5a, anterior subsector of area F5; TEs, subsector of area TE in the anterior Superior Temporal Sulcus. **(B)** Schematic flow chart of visual 3D information. Dark boxes indicate areas of the dorsal visual stream, open boxes indicate ventral stream areas, hatched boxes indicate areas selective for higher-order disparity. Boxes with question marks indicate unknown areas.

Visual object analysis in natural conditions is computationally demanding but critical for survival, hence the primate brain devotes considerable computing power to solve this problem. Lesion studies in monkeys (Ungerleider and Mishkin, 1982) and patients (Goodale et al., 1991) have demonstrated that the visual system beyond primary visual cortex consists of two subdivisions, a ventral stream directed toward the temporal cortex for object recognition and categorization, and a dorsal stream directed to the parietal cortex for spatial vision and the planning of actions (Figure 1A). Since primates not only recognize and categorize objects, but also grasp and manipulate those objects, it comes as no surprise that objects are processed in both the ventral and the dorsal visual stream.

The first recording experiments in the ventral stream, which is critical for object recognition, were published more than four decades ago (Gross et al., 1969), and the accumulated knowledge about the properties of individual neurons has spurred the development of a large number of computational models on object and shape analysis for object recognition (Riesenhuber and Poggio, 1999; Poggio and Ullman, 2013). However, neurophysiological evidence for the visual analysis of objects in the dorsal stream has only recently emerged, and biologically-plausible models for the dorsal stream are scarce (Fagg and Arbib, 1998; Molina-Vilaplana et al., 2007). Robots have to interact with objects in an unpredictable environment, and artificial vision systems that operate based on principles used by the primate dorsal stream areas could undoubtedly advance the fields of computer vision and robotics (Kruger et al., 2013). To stimulate the interaction between neurophysiology and computational modeling, it is important to review recent progress in our understanding of the neural representation of object shape in the primate dorsal visual stream.

Objects contain both two-dimensional (2D: e.g., contour, color, texture) and three-dimensional (3D: e.g., orientation in depth and depth structure) information. Originating in primary visual cortex, at least three different pathways are sensitive to depth information (Figure 1B). The MT/V5–MST/FST pathway is primarily involved in the visual analysis of moving stimuli and ego-motion, and FST neurons are selective for three-dimensional shape defined by structure-from-motion (Mysore et al., 2010). The ventral pathway V4–TEO–TEs builds a very detailed representation of the depth structure of objects, and finally the V3A–CIP–AIP–F5a pathway analyses object shape for grasping and manipulation. These pathways should not be regarded as entirely separate entities, since numerous interactions between them exist at different levels in the hierarchy. Rather, each pathway has its own specialization and can function independently of the other pathways.

In this review we will focus on the properties of individual neurons in the parietal and frontal cortex, the hierarchy of cortical areas that links early visual areas to the motor system, and the relation between neuronal firing and behavior. Neurons in other parietal areas, such as area 5 in the medial bank of the IPS (Gardner et al., 2007) and area V6A in the medial parieto-occipital cortex (Fattori et al., 2012) also respond selectively to objects of different sizes and orientations. However, the role of these neurons in computing object shape to guide the preshaping of the hand during grasping is less clear at present. We will first discuss the coding of two-dimensional (2D) shape in areas LIP and AIP, the network of areas involved in processing three-dimensional (3D) shape investigated with fMRI, and finally the single-cell properties of neurons involved in 3D shape coding in the dorsal stream.

## Two-dimensional shape selectivity in the dorsal visual stream

The first report of shape selectivity in the dorsal stream was a study by Sereno et al. (Sereno and Maunsell, 1998) in the lateral intraparietal area (LIP), an area in posterior parietal cortex (Figure 1) traditionally associated with eye movement planning and visual attention (Colby and Goldberg, 1999; Andersen and Buneo, 2002). In this study, many LIP neurons showed clear selectivity for simple two-dimensional (2D) shapes appearing in the receptive field (RF) in the absence of any eye movements. However, size and position invariance—two properties that are believed to be essential for genuine shape selectivity—were only tested in a small number of neurons. A more recent study (Janssen et al., 2008) confirmed the presence of shape-selective responses in LIP. However, a more systematic test of size and position invariance revealed that LIP neurons rarely exhibit these properties. In many cases shape-selective responses arose because of accidental interactions between the shape and the RF, such as a partial overlap. The RF structure of these LIP neurons was frequently inhomogeneous with multiple local maxima, and could even depend on the stimulus and the task: for example the RF tested with small shapes could be different from the RF tested with saccades. The lack of tolerance to changes in stimulus position in LIP neurons represented the first evidence that the shape representation in the dorsal stream is fundamentally distinct from the shape representation in the ventral visual stream, which is characterized by shape selectivity *and* tolerance of the shape preference to changes in stimulus position.

Just anterior to LIP lies area AIP (Figure 1), an area known to be critical for object grasping (Gallese et al., 1994; Murata et al., 2000; Baumann et al., 2009). Romero et al. (2012), recorded in area AIP using 2D images of familiar (e.g., fruits) and unfamiliar (tools) objects. Almost all AIP neurons showed significant selectivity to these images of objects, but subsequent testing with silhouettes and outline stimuli revealed that this selectivity was primarily based on the contours of the images. A follow-up study (Romero et al., 2013), demonstrated that for most AIP neurons, the presence of binocular disparity in these images was not necessary, and that a population of AIP neurons represents primarily relatively simple stimulus features present in images of objects, such as aspect ratio and orientation.

**Figure 2:**
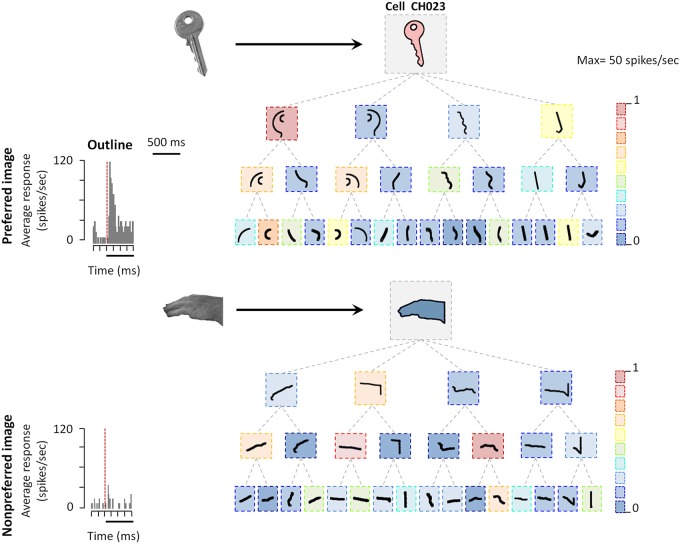
**Coding of shape features in AIP**. Example AIP neuron responding to an image of an object and to fragments of image contours. In each box the stimulus is illustrated, the color of each box represents the normalized firing rate of the neuron to that stimulus (maximum response was 29 spikes/s). Top row: intact object contour, second row: the four fragments derived from subdividing the object contour into four fragments along the main axes of the shape (four-fragment stimuli). Third and fourth row: eight- and 16-fragment stimuli. Each contour fragment is connected to the stimulus from which it was derived. The original object images from which the contours were derived are illustrated on the left side (arrows pointing to their respective contour stimuli). All stimuli were presented at the center of the RF. Reproduced with permission from (Romero et al., 2014).

The observation of neural selectivity for shape contours in AIP does not allow determining which shape features are being extracted by AIP neurons. For example, is the entire contour necessary or are parts of the shape contour (possibly corresponding to grasping affordances) sufficient to evoke AIP responses? Romero et al. (2014) used a systematic stimulus reduction approach, in which outline stimuli were fragmented into 4, 8, or 16 parts, the latter measuring merely 1–1.5°. Following previous studies in the ventral visual stream (Tanaka, 1996), the authors determined the minimal effective shape feature as the smallest fragment to which the neural response was at least 70% of the response to the intact outline. The example AIP neuron illustrated in Figure 2 responded strongly to the outline of a key but not to the outline of a monkey hand. However, some of the smallest fragments in the test still elicited robust responses in this neuron. Hence although AIP is thought to be involved in extracting grasping affordances, the AIP responses were mainly driven by very simple shape features, but not by object parts that can be grasped (Figure 2). Similar to previous observations in neighboring area LIP, the fragment selectivity depended strongly on the spatial position of the stimulus, since even small position shifts (2.5°) evoked radically different responses and therefore a very different shape selectivity. Basic orientation selectivity or differences in eye movements could not explain the fragment responses. These results suggest that AIP neurons may not extract grasp affordances. Future studies should determine how the 2D-shape representation changes in ventral premotor areas.

## A network of cortical areas sensitive to the depth structure of objects

**Figure 3:**
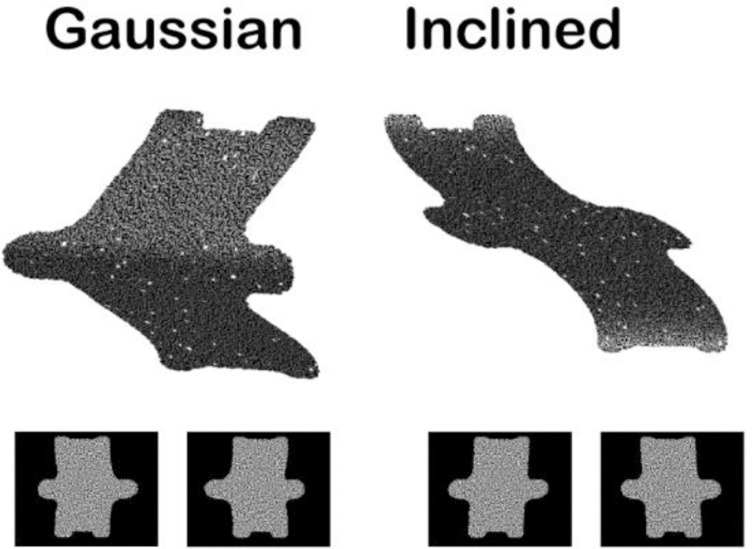
**Example random dot stereograms**. The monocular images are illustrated below a 3D rendering of two depth stimuli (Gaussian depth profile and Inclined depth profile).

The depth structure of objects (i.e., flat, convex, or concave) can be specified by a large number of depth cues such as motion parallax, texture gradients, and shading. Many studies investigating the neural basis of 3D object vision have used random dot stereograms (Figure 3), in which depth information is exclusively defined by the gradients of binocular disparity, for obvious reasons. First of all, binocular disparity is the most powerful depth cue (Howard and Rogers, 1995): even when present in isolation, disparity evokes a very vivid percept of depth that is—in contrast to motion parallax or shading—unambiguous with respect to the sign of depth (near or far, convex or concave). Moreover, physiologists are particularly keen on this type of stimuli because one can easily determine whether a neuron (or even a cortical area in the case of fMRI) is responding to the depth from disparity in the stimulus and not to other stimulus features: simply presenting the stimulus to one eye only removes all depth information in the stimulus while preserving shape and texture (Janssen et al., 1999; Durand et al., 2007). In contrast, for other depth cues such as texture gradients, determining which aspect of the stimulus the neuron responds to requires numerous control stimuli. Finally, stereograms also allow precise behavioral control: monkeys and humans can be trained to discriminate depth in stereograms, and varying the percentage of correlation between the dots in the images presented to the left and the right eye (disparity coherence) furnishes a parametric manipulation of the strength of the depth stimulus which can then be related to behaviorial performance.

A series of functional imaging and single-cell studies in monkeys (Janssen et al., 2000a; Durand et al., 2007; Joly et al., 2009) as well as imaging experiments in humans (Georgieva et al., 2009) have suggested that many cortical areas located in both the dorsal and the ventral visual stream are sensitive to the depth structure of objects (Figure 1A). This network contains AIP which, as mentioned earlier, is known to be critical for grasping (Gallese et al., 1994; Murata et al., 2000), a region (TEs) in the inferior temporal cortex (ITC), the end-stage of the ventral visual stream and critical for object recognition, and a subsector of the ventral premotor cortex (area F5a, Joly et al., 2009). The observation that not only ventral stream but also dorsal stream areas are processing 3D object information came as no surprise, since many years before these imaging studies, investigations in a patient with a ventral stream lesion had already indicated that object grasping can be intact while object recognition is severely impaired (Goodale et al., 1991; Murata et al., 2000). The object analysis required for object grasping was presumably performed by her intact dorsal stream areas (Murata et al., 2000; James et al., 2003).

## Single-cell studies in the dorsal visual stream on the visual analysis of 3D structure

fMRI can identify regions that are activated more by curved surfaces than by flat surfaces, but a detailed understanding of the neuronal selectivity in these areas requires invasive electrophysiological recordings of single neurons. Early in the hierarchy of the dorsal visual stream, the Caudal Intraparietal area (CIP) has been studied using inclined planar surfaces in which depth was defined by binocular disparity and/or texture gradients (Tsutsui et al., 2002). CIP neurons can signal the 3D-orientation (the tilt) of large planar surfaces when either disparity or texture gradients are used as a depth cue (i.e., cue invariance). A more recent report suggests that CIP neurons can also be selective for disparity-defined concave and convex surfaces (Katsuyama et al., 2010). Rosenberg et al. (2013) showed that individual CIP neurons jointly encode the tilt and slant of large planar surfaces. In view of the anatomical connections of CIP, which run along the lateral bank of the IPS toward area AIP (Nakamura et al., 2001), and more recent preliminary monkey fMRI findings (Van Dromme and Janssen, unpublished observations), CIP could be an important—but not the only (Borra et al., 2008)—input area for AIP. However, since reversible inactivation of area CIP does not cause a grasping deficit (Tsutsui et al., 2001) but sometimes a perceptual deficit in the discrimination of tilt and slant, the role of area CIP in computing 3D object shape for object grasping remains largely unknown.

**Figure 4:**
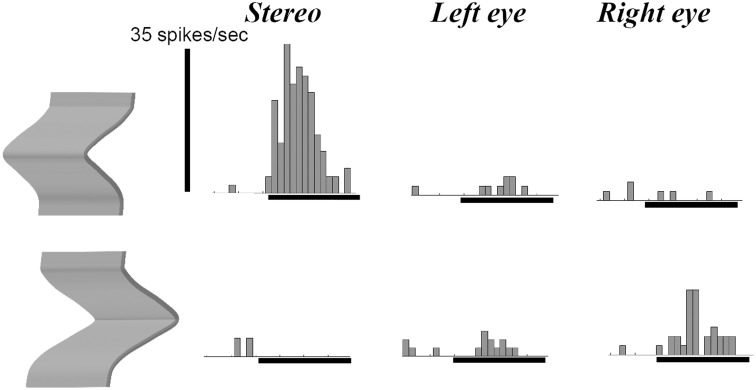
**Example neuron recorded in area AIP responding selectively to the depth structure of surfaces**. The top row shows the peristimulus-time histograms (PSTHs) of the responses to a convex depth profile, the bottom row the responses to a concave depth profile. Stereo: binocular presentation with disparity. Left eye: monocular presentation to the left eye. Right eye: monocular presentation to the right eye. The horizontal line below the PSTHs indicates the duration of stimulus presentation (800 ms). This neuron preserved its selectivity across positions in depth (data not shown).

Previous studies in area AIP had reported object-selective responses in this area (Murata et al., 2000) but it was unclear whether these neurons encoded differences in 3D structure, 2D contour, orientation or any other feature that differed between the objects used in those experiments. Srivastava et al. (2009) recorded single-cell activity in the AIP of awake fixating rhesus monkeys using disparity-defined curved surfaces. A large proportion of AIP neurons responded selectively to concave and convex surfaces that had identical contours, as illustrated by the example neuron in Figure 4. This neuron fired vigorously when a convex surface was presented, but not at all when the surface was concave, a selectivity which could not be accounted for by the responses to the monocular presentations. Since this neuron preserved its selectivity across positions in depth (data not shown), the neuron must have responded to a change in binocular disparity along the surface of the stimulus, i.e., higher-order disparity selectivity. The same study observed that the neuronal properties in AIP were markedly different from the ones in TEs: AIP neurons fired much faster to the presentation of curved surfaces (a population latency of 60–70 ms in AIP compared to 90–100 ms in TEs), but appeared less sensitive to small differences in 3D structure, including the sign of curvature: while TEs neurons frequently showed similar responses to curved surfaces with different degrees of curvedness (provided they had the same sign, i.e., either convex or concave) and large response differences when the sign of curvature changed (even for very slightly curved surfaces), the response of AIP neurons declined monotonically as the degree of curvedness was decreased. These observations were the first demonstration that a distinct representation of the depth structure of objects exists in the dorsal visual stream. A follow-up study (Theys et al., 2012b), showed that the large majority of the AIP neurons are primarily sensitive to the disparity gradients on the boundary of the stimulus but largely ignore the depth structure information on the surface, which represents another difference with TEs neurons.

The ventral premotor cortex (PMv) represents the main target of area AIP (Borra et al., 2008). Reversible inactivation of PMv produces a grasping deficit that is highly similar to the one seen after AIP inactivation (Gallese et al., 1994; Fogassi et al., 2001). However, recent studies have provided functional and anatomical evidence suggesting that PMv is not a homogeneous area. A monkey fMRI study (Joly et al., 2009) showed that a subsector of PMv, area F5a, is more activated by curved surfaces than by flat surfaces at different positions in depth, similar to AIP. This activation was located in the depth of the more anterior part of the inferior ramus of the arcuate sulcus. Recent anatomical studies (Belmalih et al., 2009; Gerbella et al., 2011) described differences in the cytoarchitectonics and anatomical connectivity between area F5a and the surrounding subsectors of PMv: F5a does not project directly to primary motor cortex, but does so through its connections with F5p. Moreover, F5a is more strongly connected to the parietal (AIP) and prefrontal (areas 46 and 12) cortex. Based on the anatomical connectivity of F5a, Gerbella et al. (2011) coined the term “pre-premotor cortex” for area F5a, indicating that this subsector of PMv could represent a stage upstream from the more widely studied F5p and F5c sectors of PMv.

**Figure 5:**
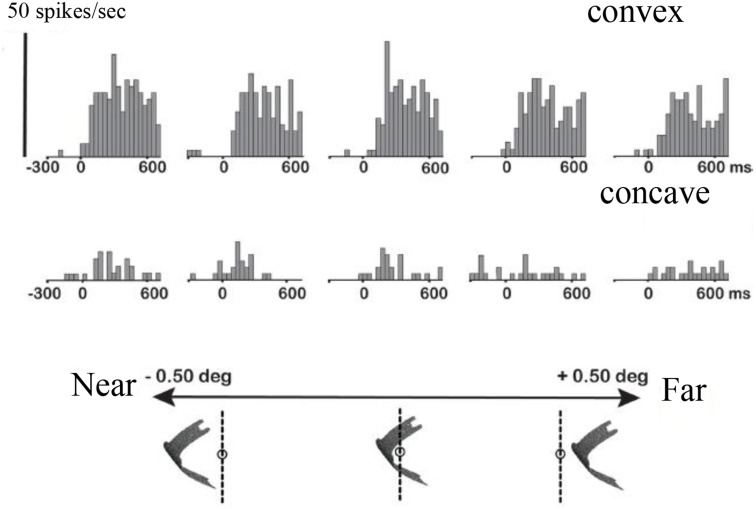
**Higher-order disparity selective F5a neuron**. The PSTHs in the top row illustrate the responses of a single F5a neuron to a convex depth profile at five different positions in depth, the bottom row the responses to a concave depth profile at the same positions in depth. Below the PSTHs is a schematic illustration of the stimulus presentation indicating the positions in depth. Reproduced with permission from (Theys et al., 2012a).

Guided by a monkey fMRI study (Joly et al., 2009), Theys et al. (2012a) targeted the F5a subsector with microelectrode recordings to investigate in detail to what extent this region differs functionally from the other subsectors of PMv. Accurately predicted by fMRI, neurons selective for disparity-defined curved surfaces were located in F5a but not in surrounding regions of PMv. The example neuron in Figure 5 responded to a convex depth profile but not to a concave depth profile irrespective of the position in depth of the stimulus, and monocular responses could not account for the selectivity (data not shown). Remarkably in view of its anatomical location in the premotor cortex, the responses of these F5a neurons appeared very “visual,” with robust increases in firing rate when visual stimuli (that the monkey could not grasp) appeared on a display, and with relatively short response latencies (70–80 ms) compared to 50–60 ms for AIP, which is consistent with the higher position in the cortical hierarchy of F5a compared to AIP. These strong visual responses to images presented on a display were unexpected, since previous studies (Graziano et al., 1997; Graziano and Gross, 1998) did not observe responses to images of objects presented on a display in PMv neurons with bimodal visuo-tactile responses. i.e., responses to tactile stimulation of the face or hand and to visual presentation of objects near the face or hand.

These observations raised the question whether F5a could still be considered part of PMv. To that end, (Theys et al., 2012a)—after having established higher-order disparity selectivity in a cluster of F5a neurons—recorded from the same neurons while the animal was grasping objects in the light (i.e., visually-guided grasping) and in the dark (i.e., memory-guided grasping). Surprisingly, almost all F5a neurons selective for disparity-defined depth structure were also active in the light when the monkey was grasping objects that did not resemble the random-dot stereograms. However, most of these F5a neurons were virtually silent when the animal was grasping the same objects in the dark. Hence, in contrast to earlier reports stating that all PMv neurons remain active during grasping in the dark (Raos et al., 2006), F5a neurons selective for the depth structure of objects are mostly visual-dominant. These results do not imply that all F5a neurons are visual-dominant, since multi-unit recordings of clusters of 3D-structure selective neurons revealed strong grasping-related activity in the dark. The implication of these findings is that (visual-dominant) 3D-structure selective F5a neurons are co-localized and strongly connected with visuomotor and motor-dominant neurons that remain active in the absence of visual information. Such functional clusters may form neural modules in which visual object information is mapped onto motor commands. The presence of activity during grasping in the dark implies that F5a is effectively a subsector of PMv, which differs from the other subsectors of PMv by the presence of visual-dominant responses during grasping and selectivity to the depth structure of objects.

**Figure 6:**
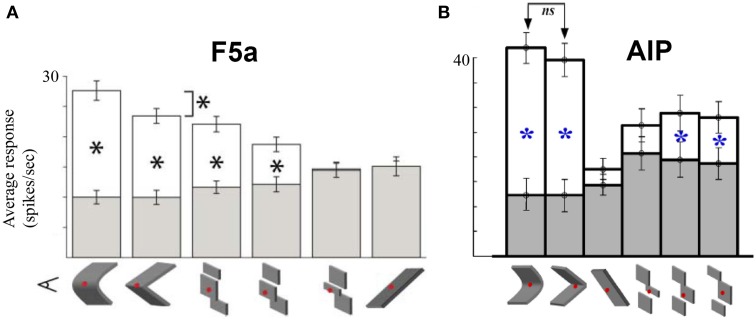
**Comparison between AIP and F5a. (A)** Average responses of a population of F5a neurons to various approximations of the smoothly curved surfaces (on the left). White bars indicate the responses to the preferred depth profile, filled bars the responses to the non-preferred depth profile. Below the bar graph are schematic illustrations of the various approximations: linear, three different discrete approximations, and first-order (tilted plane). The asterisks indicate significant differences in response. **(B)** Average responses of a population of AIP neurons to the same stimuli. Same conventions as in **(A)**. Adapted with permission from (Srivastava et al., 2009).

The previous studies suggest that the hierarchy of dorsal stream cortical areas involved in 3D-shape processing is largely serial. Although most likely an oversimplification (e.g., the role of feedback connections is unknown and ignored here), the CIP–AIP–F5a serial chain of areas provides a unique opportunity to investigate how the 3D-shape representation changes along the dorsal pathway so that the underlying computations might be revealed. In a first attempt to address this question, Theys et al. (2013) recorded in F5a and AIP in the same animals during visual presentation of 3D surfaces, various approximations of these surfaces and during object grasping. The sensitivity for depth structure was measured by plotting the average responses of a population of neurons to curved surfaces with varying degrees of the disparity variation (from very convex, over almost flat to very concave surfaces). Although interindividual differences between the two animals were present, the sensitivity functions were virtually identical in F5a and AIP. Furthermore, testing F5a neurons with planar (i.e., least-square) and discrete approximations of the smoothly curved surfaces showed again strong similarities between AIP and F5a (Figure 6): the majority of neurons in AIP and F5a was also selective for discrete approximations of the convex and concave surfaces consisting of three separate planes in depth, but in F5a, the linear approximation evoked significantly less responses compared to the smoothly curved surfaces. Finally, AIP neurons encoding depth structure from disparity were also tested during object grasping, and similar to F5a, most of these AIP neurons were also strongly active during grasping. The only difference between the AIP and the F5a multi-unit activity consisted of stronger and faster responses in AIP during object fixation and higher activity in F5a during the hand movement epoch before object lift, suggesting that AIP neurons are mainly active during the visual analysis of the object whereas F5a neurons remain active throughout the trial. Overall, the representation of depth structure in premotor area F5a was highly similar to that in parietal areas AIP, which makes it difficult to identify the computations that take place between these two different stages in the dorsal stream shape hierarchy. Future studies may be able to document the differences in the object representation between F5a and AIP using different stimuli or tasks.

The strong correspondence between depth structure selectivity and grasping responses in AIP and F5a is consistent with the hypothesis that the pre-shaping of the hand during visually-guided object grasping relies on 3D-object information in these two areas. The question still remains why the primate brain would maintain two highly similar areas that communicate by means of (metabolically expensive) long-range connections. At this point the data suggest that the neural object representation in AIP does not have to become much more elaborate to guide the hand during grasping. Furthermore, F5a can directly interact with other premotor areas such as F5p, which AIP can also access directly or indirectly through either parietal area PFG or F5a. The interpretation of “motor” activity, i.e., activity during grasping in the dark, may be crucial in this respect. Both F5a and AIP contain large numbers of neurons that are active in the dark, in the absence of visual information, which are typically not selective for disparity-defined depth structure. Traditionally, this “motor” type of activity has been interpreted as related to action planning, since the activity remains high in the delay period when the animal is waiting for the go-signal to execute the grasping action. However, the activity in the dark in AIP may have a different status: AIP neurons may receive corollary information from premotor areas about the movement planning which can be integrated with visual information during visually-guided grasping (Rizzolatti and Luppino, 2001), an idea that has never been tested. Under this hypothesis, F5a motor activity is genuinely sub-serving action planning, whereas AIP motor activity is simply a corollary discharge reflecting premotor signals.

## Conclusions

More than four decades of research have been devoted to investigations of the object representation in the ITC (for review, see Tanaka, 1996). ITC neurons respond selectively to shapes, and at the same time achieve selectivity invariance, i.e., these neurons exhibit tolerance of shape preference for stimulus transformations such as changes in retinal position, size, the visual cue defining the shape and occlusion. These neuronal properties are believed to be essential to support robust object recognition in an ever changing environment. Single-cell studies have demonstrated that neurons in TEs, a subsector of the ITC located anteriorly and therefore one of the end-stage areas of the ventral visual stream, also encode the 3D structure of surfaces (Janssen et al., 2000a,b) defined by binocular disparity and 3D orientation defined by disparity and texture (Liu et al., 2004), and TEs activity is causally related to the categorization of depth structure (Verhoef et al., 2012). Undoubtedly, a complex hierarchy of visual areas along the ventral visual stream supports the high-level 3D object representation culminating in TEs.

In the dorsal visual stream, the neural representations of depth structure can be traced from mid-level visual area CIP, which presumably receives input from early visual area V3A, until the motor system (F5a). Somewhere along this dorsal pathway, visual object representations are transformed into motor commands (grip type representations) that control the preshaping of the hand during object grasping. As outline above, inactivation studies have demonstrated that at least AIP and F5 are both important for motor control during grasping, but the role of other parietal areas such as PFG (Bonini et al., 2012) and V6A (Fattori et al., 2010) in grasping requires further study. Investigating the neural representation of object shape demands systematic stimulus manipulations (e.g., stimulus reduction), therefore visual object representations can be primarily studied in neurons that respond to images of objects (either 3D or 2D), as in AIP and F5a. Neurons in F5p and F5c, in contrast, respond selectively to real-world objects (Raos et al., 2006) but not to 3D images of objects (Theys et al., 2012a), most likely because these areas represent grip types, which are not activated by images of objects.

Numerous very basic questions remain to be addressed in future studies: for example, how do the RFs change along the dorsal pathway, which computations take place at different stages, what is the role of feedback projections? As outlined earlier, it is also unclear at present how the ventral stream division of this 3D-shape network is organized. Our comprehension of this network will only increase when studies combine electrophysiological recordings, imaging and ultimately also computational modeling.

### Conflict of interest statement

The authors declare that the research was conducted in the absence of any commercial or financial relationships that could be construed as a potential conflict of interest.
